# P-569. Epidemic Preparedness and Vaccine Equity: Access for All

**DOI:** 10.1093/ofid/ofaf695.784

**Published:** 2026-01-11

**Authors:** John R Bassler, David T Redden, Scott Harris, Paul C Erwin, Casey L Daniel

**Affiliations:** University of Alabama at Birmingham, Birmingham, AL; Edward Via College of Osteopathic Medicine, Auburn, Alabama; Alabama Department of Public Health (ADPH), Montgomery, Alabama; University of Alabama at Birmingham, Birmingham, AL; University of South Alabama, Mobile, Alabama

## Abstract

**Background:**

Amidst the vaccination efforts during the COVID-19 Pandemic, rollout was expanded to regional and supermarket pharmacies to ease the burden on traditional deliveries. To address low vaccine uptake, the initiative was expanded to include Black-owned barbershops and hair salons as sources for accurate information and to administer vaccines where possible. While the rollout was well-intentioned, geographical disparities of vaccination rates (VR) persisted. This report investigates the viability of expanding vaccine delivery systems to include Dollar General (DG) locations as vaccination partners to address geographic disparities in vaccine access.

**Figure 1.**
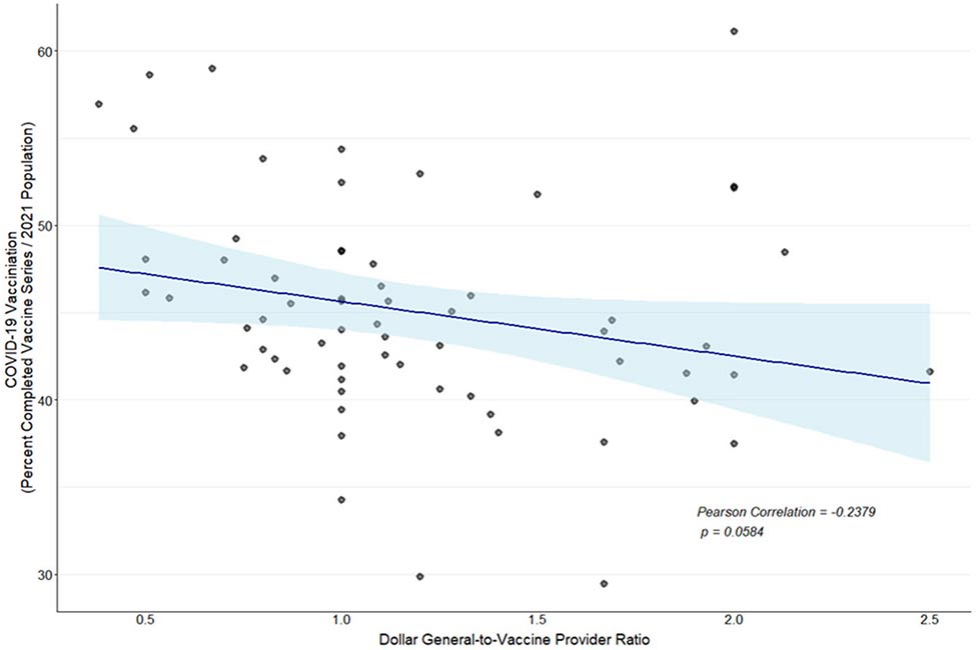
Linear trend (95% Confidence Interval) between Alabama County COVID-19 vaccination rates and ratio of Dollar General locations to COVID-19 vaccination providers

**Methods:**

With low ranking COVID-19 vaccination rates among US states, Alabama (AL) was chosen to investigate the relationship between VR, vaccine providers, and DG locations. County percentages of people who completed the primary series of COVID-19 vaccinations were sourced from the AL Department of Public Health (ADPH) COVID-19 Dashboard. COVID-19 vaccine locations (publicly provided by CDC PLACES and ADPH dashboard) and DG locations were geocoded and summarized by County. General linear models were used to assess associations with VR, including a ratio of the number of DGs and vaccination providers.

**Results:**

Among all 67 counties in AL, the median VR was 44.1% (IQR: 41.5%, 48.3%). The median DG-to-provider ratio was 1.1 (IQR: 0.9, 1.7). Components of the Social Deprivation Index were found to have an inverse relationship with VR, including lack of education (p = .002) and unemployment (p = .005). While not statistically significant, county VR decreased as the DG-to-provider ratio increased (p = .058, Figure 1). Among the five counties with the highest VR ( >55%), four out of five had DG-to-provider ratio < 1.0 (https://jbassler.shinyapps.io/AL-COVID19-DG/).

**Conclusion:**

In areas with historically low VR, or limited access to vaccine providers, DGs in these areas provide a scalable solution to expanding vaccine delivery. In addition to their distribution network, DG creates the point-of-contact needed for vaccine outreach. The strategic co-location of mobile vaccine initiatives at DGs in vulnerable communities provides a scalable method to achieve geographically equitable vaccine coverage.

**Disclosures:**

All Authors: No reported disclosures

